# Identifying socioeconomic characteristics of pregnant women aged 15–50 years diagnosed with HIV during pregnancy, the Netherlands, 2016 to 2023

**DOI:** 10.2807/1560-7917.ES.2026.31.37.2600048

**Published:** 2026-09-17

**Authors:** Colette Smit, Vita Jongen, Jeannine Nellen, Tania Mudrikova, Elisabeth van Leeuwen, Nina Schat, Rosan van Zoest, Ard van Sighem, Eline Op de Coul, Marc van der Valk, on behalf of the ATHENA observational HIV cohort

**Affiliations:** 1 Stichting HIV monitoring, Amsterdam, the Netherlands; 2 Amsterdam University Medical Centre, University of Amsterdam, Department of Infectious Diseases, Amsterdam Institute for Infection and Immunity, Amsterdam, the Netherlands; 3 Department of Infectious Diseases, Public Health Service of Amsterdam, Amsterdam, the Netherlands; 4 Department of Rheumatology, Immunology and Infectious Diseases, University Medical Centre Utrecht, Utrecht, the Netherlands; 5 Amsterdam Health and Technology Institute, Amsterdam, the Netherlands; 6 Centre for Infectious Disease Control, National Institute for Public Health and the Environment, Bilthoven, the Netherlands; 7 The members of the ATHENA observational HIV cohort are acknowledged at the end of the article

**Keywords:** HIV, pregnancy, screening programs, public health, epidemiology

## Abstract

**BACKGROUND:**

Pregnant women in the Netherlands are screened for HIV during the first trimester. Insight into characteristics of women diagnosed with HIV may help identify groups who could benefit from testing strategies.

**AIM:**

We assessed the contribution of HIV diagnoses during pregnancy to the total number of diagnoses among women in the Netherlands between 2016 and 2023 and identified socioeconomic characteristics of women with an antenatal diagnosis.

**METHODS:**

In a cross-sectional analysis, we selected women aged 15–50 years registered in the national ATHENA cohort and diagnosed with HIV in the Netherlands. Each pregnant woman with HIV was matched by year of pregnancy to three pregnant women without HIV from the general population using non-public registry data from Statistics Netherlands. Characteristics were examined using multivariable logistic regression.

**RESULTS:**

Ninety-two of 523 women with HIV were diagnosed during pregnancy. These women less often had an advanced HIV infection than non-pregnant women with HIV (22 vs. 47%). Eighty-three of 92 pregnant women with HIV were matched with 249 pregnant women without HIV. Being diagnosed during pregnancy was associated with first- (aOR: 14.1; 95% CI: 6.45–30.6) or second-generation migration background (aOR: 5.38; 95% CI:2.02–11.0), income below the poverty line (aOR: 3.46; 95% CI: 1.28–9.34) and living in a single-parent household (aOR: 3.46; 95% CI: 1.52–7.88).

**CONCLUSION:**

Antenatal HIV diagnoses accounted for a substantial part of diagnoses among women; pregnant women were less likely to present with advanced-stage disease. These findings show the importance of universal antenatal HIV screening.

**Key public health message**

**What did you want to address in this study and why?**

The most common mode of acquiring HIV for children is perinatal transmission. Therefore, timely initiation of treatment during the mother’s pregnancy is important. Pregnant women in the Netherlands are screened for HIV during the first trimester. We wanted to assess the number of HIV diagnoses during pregnancy in the Netherlands and compare pregnant women with and without HIV to explore differences in socioeconomic characteristics.

**What have we learnt from this study?**

We learnt that 18% of women diagnosed with HIV in the Netherlands received their diagnosis during pregnancy and these women were less often diagnosed with advanced-stage HIV than women who were not pregnant. Compared with pregnant women without HIV, women with an antenatal diagnosis more often had a migration background, lower income and lived in a single household.

**What are the implications of your findings for public health?**

Women diagnosed with HIV during pregnancy account for nearly one fifth of HIV diagnoses among women in the Netherlands. The study findings support the implementation of targeted prevention strategies, such as preconception counselling and repeat testing during pregnancy for women at increased risk. They also highlight the need to reconsider pre-exposure prophylaxis access for women.

## Introduction

The most common mode of HIV acquisition among children aged 0 to 15 years worldwide is perinatal transmission [1]. Perinatal transmission of HIV mainly occurs during labour and delivery, or through breastfeeding. Without intervention, the risk of perinatal transmission varies between 15% and 45% [2]. However, with the introduction of combination antiretroviral therapy (ART) the risk of perinatal transmission has been reduced to less than 1% [3,4].

Timely identification of HIV and early initiation of ART in pregnant women are therefore crucial to prevent perinatal transmission. Antenatal HIV screening is widely implemented in European countries, most commonly through an opt-out strategy [5].

In the Netherlands, the estimated number of women with an undiagnosed HIV infection in 2024 was 265 (95% confidence interval (CI): 210–325) and 4,619 women had been diagnosed and linked to care in 2024. Between 2022 and 2024, 58% of women newly diagnosed with HIV were diagnosed at a late stage [6]. In January 2004, the Netherlands introduced standardised opt-out HIV antibody testing for all pregnant women during the first trimester [7,8]. Participation rates for antenatal HIV screening are above 99% in the Netherlands and 77% of women screened are in their first trimester [9]. Previous research has demonstrated the effectiveness of this programme in identifying pregnant women with HIV and preventing vertical transmission [7]. The participation rate in the Dutch antenatal screening programme is high and the HIV prevalence detected though the programmme was 0.05% and stable over the past few years [10]. Women diagnosed with HIV during pregnancy are referred to one of the HIV treatment centres for antenatal HIV care. This care is provided by a multidisciplinary team, including an HIV treating physician, an obstetrician and a paediatrician. This approach ensures optimal counselling focused on the adjustment to an HIV diagnosis, initiation of ART and preparation for the post-partum period, including informed decision-making regarding infant feeding options [11].

Gaining insight into the sociodemographic and socioeconomic characteristics of women living in the Netherlands who are newly diagnosed with HIV during pregnancy may help identify groups who could benefit from adapted targeted HIV testing strategies. This study has two main objectives: (i) to quantify the proportion of women diagnosed with HIV during pregnancy of all new HIV diagnoses among women aged 15–50 years (2016–2023), and (ii) to compare pregnant women with and without HIV to identify differences in sociodemographic and socioeconomic characteristics between women diagnosed with HIV during pregnancy and pregnant women without HIV using data from the AIDS Therapy Evaluation in the Netherlands (ATHENA) cohort and registry data from Statistics Netherlands.

## Methods

### Study design

We conducted a cross-sectional secondary analysis using individual data from the ATHENA cohort and non-public microdata from Statistics Netherlands. Briefly, HIV care in the Netherlands is provided by 23 designated treatment centres. The Dutch HIV Monitoring Foundation (Stichting HIV monitoring (SHM)) is tasked by the Ministry of Health, Welfare and Sports to monitor and report on all aspects of HIV care for people with HIV in the Netherlands. Data collection was initiated in 1998 and data have been prospectively collected in the ATHENA cohort, which represents over 97% of all people with HIV in care in the Netherlands [12].

Statistics Netherlands is an independent organisation that collects, processes and publishes reliable statistical data on residents of the Netherlands. The Statistics Netherlands Act constitutes the legal basis for Statistics Netherlands, which adheres to the European Union’s General Data Protection Regulation.

For the present study, ATHENA cohort data were combined with data from Statistics Netherlands using a probabilistic approach based on date of birth, four digits from the postal code of last known residence and sex assigned at birth. Participants from the ATHENA cohort provided consent for the use of their data for data linkage purposes and information about active data linkages is available on the SHM website. The Remote Access environment of Statistics Netherlands is only available for researchers authorised by SHM and Statistics Netherlands. All output from the Remote Access environment is independently verified by Statistics Netherlands to ensure data cannot be traced back to individuals.

### Study population

We included all women aged 15–50 years diagnosed with HIV in the Netherlands between 2016 and 2023 from the ATHENA cohort. Women with a known HIV diagnosis before migration to the Netherlands were excluded. Women were classified as pregnant or not at the time of diagnosis. First, women with an HIV diagnosis during pregnancy were compared to non-pregnant women with an HIV diagnosis to quantify the proportion of HIV diagnoses during pregnancy to all HIV diagnoses.

Second, pregnant women with an HIV diagnosis during pregnancy were compared to pregnant women without HIV in the general population (the control group). To assess differences in sociodemographic and socioeconomic characteristics, pregnant women in the general population were identified using data from Statistics Netherlands (2016–2022). These women were identified by their declaration of maternity care costs, which included care provided for live births as well as terminated pregnancies. These data were available up to 2022. To minimise imbalance between the number of pregnant women with and without HIV, each pregnant woman with HIV was randomly matched with three pregnant women without HIV from the general population by year of pregnancy to account for potential effects related to the COVID-19 pandemic on socioeconomic characteristics. No additional variables were used for matching; instead, relevant covariates were included in the multivariable analyses. Women were not matched on age since all women were pregnant, representing a specific age range. Other available variables such as socioeconomic and demographic variables were also not included as these were the variables of interest.

### Study variables

Calendar year (1 January–31 December of a given year) of diagnosis was grouped into broader categories since the small number of women diagnosed with HIV during pregnancy in some years, combined with pregnancy status, could potentially allow for the identification of individuals. Data were aggregated to minimise this risk. Clinical data at the time of HIV diagnosis included age, CD4^+^ cell count and percentage, presence of AIDS-defining clinical events, a negative HIV test before diagnosis and diagnosis setting. Stage of HIV at diagnosis was defined as: (i) advanced (CD4^+^ cell count < 200 cells/mm^3^ and/or an AIDS-defining event); (ii) late (CD4^+^ cell count 200–350 cells/mm^3^); or (iii) established/recent (CD4^+^ cell count > 350 cells/mm^3^ or evidence of a recent HIV infection based on a documented negative HIV test within the past 12 months). If case information was not available on CD4^+^ cell counts, the presence of AIDS-defining events or a recent test, stage of HIV diagnosis was defined as unknown. Information on earlier pregnancies was only collected for pregnant women with HIV.

Statistics Netherlands provided detailed individual-level sociodemographic and socioeconomic information, including education level, migration background, employment status, household composition, household income, social welfare use and urbanisation level of the municipality, all in the year of conception. Level of education was categorised as primary (completed pre-vocational secondary education, first 3 years of senior general secondary education), secondary (completed vocational education, senior general secondary education or pre-university level) or college/university. Migration background was defined as no migration background (a person with both parents born in the Netherlands), first-generation migration background (a person born abroad) or second-generation migration background (a person born in the Netherlands with at least one parent born abroad). Household income was defined according to the social minimum or poverty line (the minimal amount of financial resources required to achieve a minimally acceptable lifestyle). The social minimum is determined and adjusted biannually by the Ministry of Social Affairs and Employment [13]. An individual’s income was categorised as below the poverty line if their household income was lower than 120% of the social minimum, which determines if an individual is eligible for benefits in the Netherlands. Urbanisation level was used to categorise municipalities according to the population density into high (very urban and urban with ≥ 1,500 addresses per square kilometre) and middle/low (not urban/not rural, and rural with < 1,500 addresses per square kilometre).

### Statistical analysis

The annual number of HIV diagnoses among women within the reproductive lifespan (aged 15–50 years) in the Netherlands were described and the proportion of women with a positive HIV antenatal test calculated. Characteristics at the time of HIV diagnosis were described using a Pearson chi-square test for categorical variables and Mann-Whitney test for continuous variables. Missing values were analysed as a separate category. Sociodemographic and socioeconomic characteristics were compared between pregnant women with an HIV diagnosis during pregnancy and pregnant women without an HIV diagnosis using adjusted odds ratios (aOR) obtained from multivariable logistic regression. Variables included in the multivariable logistic regression were: age, migration background, urbanisation level, household income, household composition, education, employment status and household income source. Multivariable models were built using backward selection and characteristics with an univariable p value < 0.20 were included in the multivariable models. As income was correlated with education, receiving social welfare and employment status, we included two multivariable logistic regression models: one included only household income, and a second excluded household income but included education, receiving social welfare and employment status.

The results presented are based on calculations carried out by SHM, project number 8944 using non-public microdata from Statistics Netherlands and Vektis C.V. Analysis was carried out using SPSS version 25 (IBM Corp., Armonk, United States (US)) and SAS version 9.4 (SAS Institute Inc., Cary, US).

## Results

### Number of new HIV diagnoses

**Figure 1:**
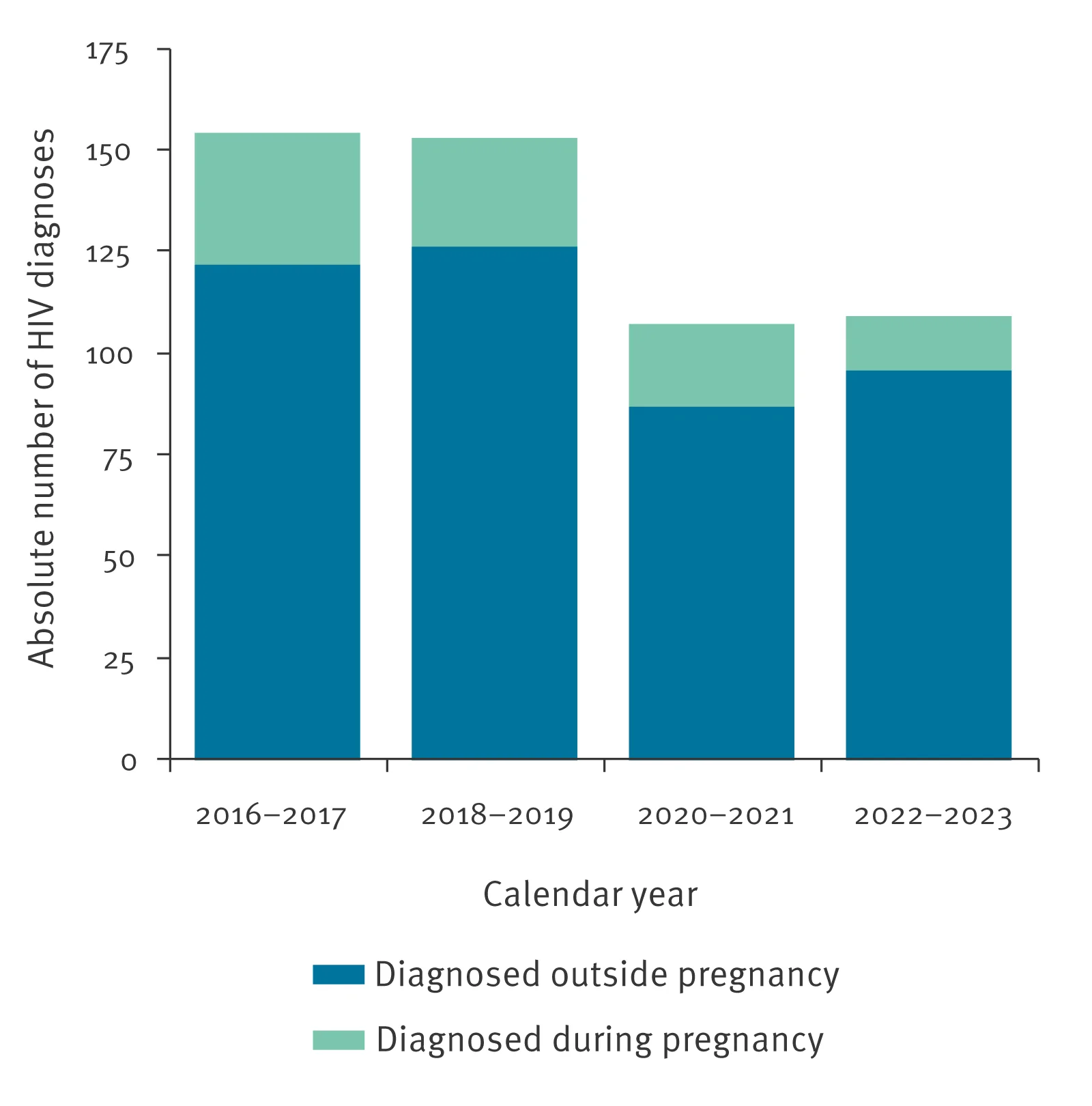
Annual number of HIV diagnoses among women aged 15–50 years, stratified by diagnosis during and outside pregnancy, the Netherlands, 2016–2023

Between 2016 and 2023, 523 women aged 15–50 years were newly diagnosed with HIV in the Netherlands and registered in ATHENA. The annual number of diagnoses fluctuated between 82 and 49 diagnoses per year (Figure 1). Of these, 92 (18%) were diagnosed with HIV during pregnancy and 431 outside pregnancy. For five women, pregnancy screening was the documented setting for testing, but pregnancy status could not be confirmed. At the time of diagnosis, pregnant women were younger (median age 30 years; interquartile range (IQR): 27–35 years) compared with non-pregnant women (median age 37 years; IQR: 28–42 years, p < 0.001, Table 1).

**Table 1:** Characteristics of women aged 15–50 years at the time of an HIV diagnosis, the Netherlands, 2016–2023

Characteristics	Non-pregnant with HIV				Diagnosed with HIV during pregnancy		p value	All	
	n			%	n	%		n	%
Total	431			82	92	18	NA	523	100
Age at diagnosis (years), median age (IQR)	37 (28–42)				30 (27–35)		< 0.001	35 (28–41)	
CD4^+^ cell count (cells/mm^3^), median (IQR)	284 (89–519)				330 (210–470)		0.08	300 (120–510)	
CD4^+^ percentage, median (IQR)	19 (10–27)				23 (16–29)		0.06	20 (11–27)	
Earlier negative HIV test	187		43		53 (58)		0.01	240	46
Time to HIV diagnosis since negative test (years), median (IQR)	3.6 (1.3–7.5)				2.9 (1.0–6.7)		0.40	3.5 (1.2–7.3)	
Negative HIV test within 12 months of diagnosis									
No	142		33		40	43	0.07	182	35
Yes	49		11		13	14		62	12
Unknown^a^	240		56		39	42		279	53
Setting of HIV testing									
Pregnancy screening programme	5		1		75	82	< 0.001	80	15
General practice	159		37		3	3		162	31
Sexual health centres/municipal public health service	49		11		4	4		53	10
Hospital^b,c^	188		43		7	8		195	37
Other	15	3			0			15	3
Unknown^a^	15	3			3	3		18	3
Gravidity									
First pregnancy	NA				32	34	NA	NA	
Previous pregnancy					58	63			
Missing^d^					2	2			
Parity									
Nullipara	NA				45	49	NA	NA	
Primi- or multipara					45	49			
Missing^d^					2	2			

IQR: interquartile range; NA: not applicable.

^a^ Unknown refers to a specific category indicating that the status was actively assessed but could not be determined.

^b^ Ten non-pregnant women were diagnosed by a gynaecologist, obstetrician or fertility care in the hospital.

^c^ Five pregnant women were diagnosed by a gynaecologist, obstetrician or fertility care in the hospital.

^d^ Missing refers to variables for which no information was recorded.

### Clinical stage at time of HIV diagnosis

Of the 523 women newly diagnosed with HIV, 240 had a previous negative HIV test result, with 12% tested within 12 months before diagnosis (Table 1). Women diagnosed during pregnancy more often had a previous negative HIV test (58%) than women diagnosed outside pregnancy (43%; p = 0.01). Interestingly, 2 of 92 women diagnosed during pregnancy had a previous negative HIV test during the same pregnancy. For five women, their previous negative test result was within 6 months before conception.

Median CD4^+^ cell count at the time of HIV diagnosis was 330 cells/mm^3^ (IQR: 210–470) for pregnant women and 284 cells/mm^3^ (IQR: 89–519) for non-pregnant women. When looking at CD4^+^ percentages instead of CD4^+^ cell count, both the median and IQR were found to be higher among pregnant women (23%; IQR: 16–29) than non-pregnant women (19%; IQR: 10–27) (p = 0.06).

**Figure 2:**
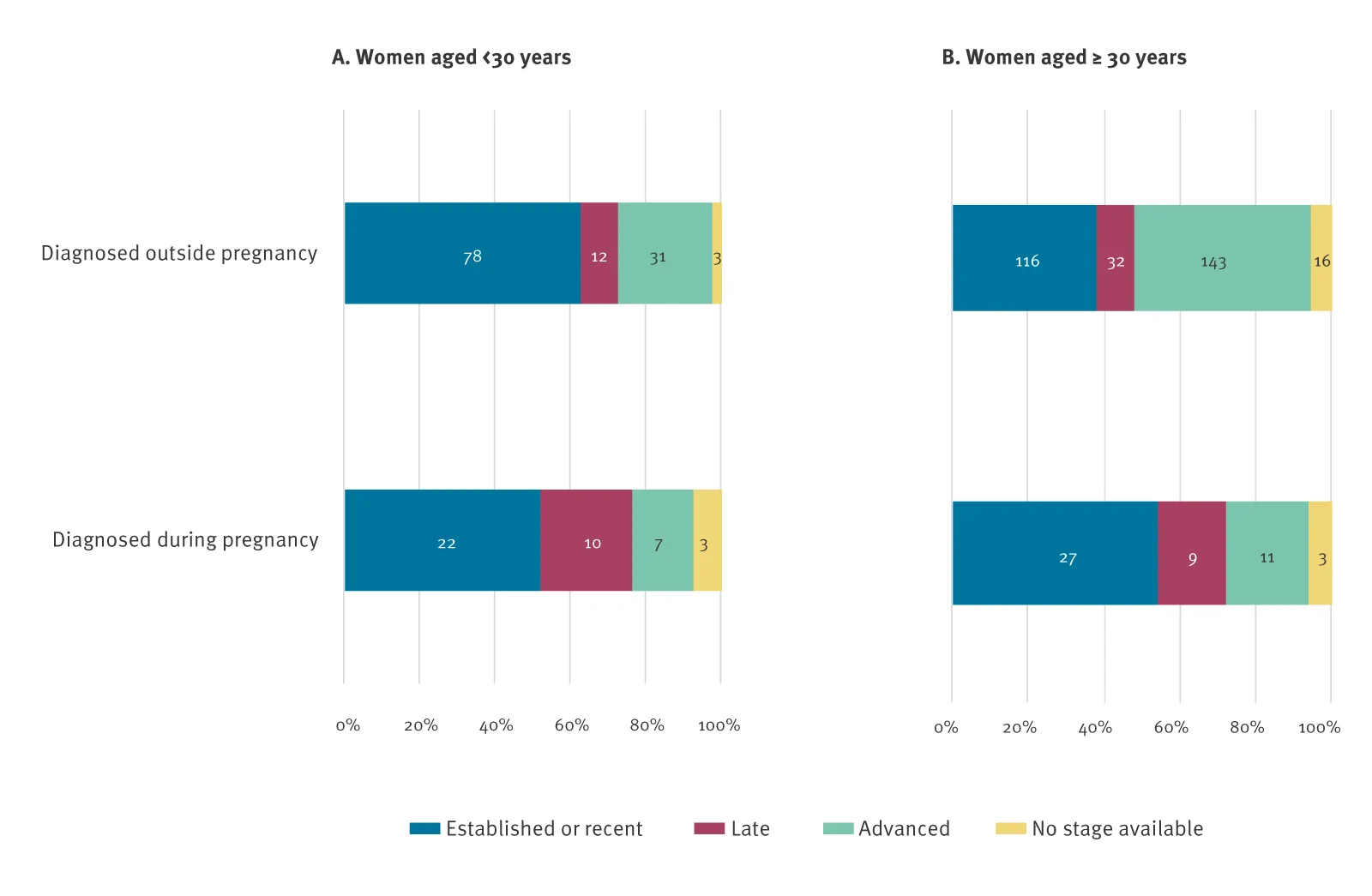
Stage of HIV at diagnosis among women aged 15–50 years diagnosed during and outside pregnancy, by age group, the Netherlands, 2016–2023

Overall, the proportion of included women diagnosed with advanced-stage HIV was 37% (192/523). A twice as high proportion of non-pregnant women had advanced-stage HIV at diagnosis (174/431; 40%), compared with pregnant women (18/92; 20%). When stratified by age, the difference in advanced-stage HIV was less pronounced among women aged younger than 30 years (Figure 2A) compared to pregnant women aged 30 years or older. Of the pregnant women aged 30 years or older, 54% had an established or recent HIV stage and 18% had late-stage HIV. Twenty-two percent (11/50) of pregnant women and 47% (143/307) of non-pregnant women aged 30 years or older presented with advanced-stage HIV (p = 0.02) (Figure 2B). Notably, among women aged 30 years or older with advanced HIV, median age was 40 years (IQR: 36–45) for non-pregnant women and 36 years (IQR: 32–39) for pregnant women (p = 0.01).

The numbers in the bars represent the absolute number of women.

### Setting of HIV diagnosis

Information on the setting of HIV diagnosis was available for 505 of the 523 women (97%). Among pregnant women, 82% (n = 75) were diagnosed through the pregnancy screening programme. Five pregnant women (5%) were diagnosed at a hospital by a gynaecologist, obstetrician or in fertility care. In contrast, 2% of non-pregnant women were diagnosed at a hospital by a gynaecologist, obstetrician or in fertility care (Table 1).

### Earlier pregnancies

Of 92 women diagnosed with HIV during pregnancy, 32 were pregnant for the first time and 58 had a previous pregnancy (gravidity > 1); for two women, information on earlier pregnancies was missing (Table 1). Of the 58 women who were diagnosed with HIV during pregnancy and who had a previous pregnancy, 38 (66%) reported a previous negative HIV test result and 20 (34%) did not have information on a previous HIV negative test. Of the 32 women who were pregnant for the first time, 13 (41%) had a previous negative HIV test result. This was significantly lower (p = 0.02) than for women with a previous pregnancy.

### Comparison with pregnant women without HIV

We were able to combine data for 83 of 92 (90%) women diagnosed with HIV during pregnancy with data from Statistics Netherlands. These women were matched with 249 pregnant women without HIV (Table 2). Pregnant women without HIV were more often aged between 25 and 35 years (73%) compared with women diagnosed with HIV during the pregnancy (54%). When women diagnosed with HIV during pregnancy were compared with the control group, they more often had a first-generation migration background (70% vs 20%), while 14% (n = 12) had a second-generation migration background and 16% (n = 13) had no migration background. Furthermore, women diagnosed with HIV during pregnancy more frequently had an income below the poverty line when compared to pregnant women without HIV (39% vs 6%) and lived in a single or single-parent household (36% vs 13%) (Table 2).

**Table 2:** Socioeconomic and demographic characteristics of pregnant women stratified by HIV status, the Netherlands, 2016–2023 (n = 332)

Characteristics	Total (n = 332)		Pregnant women without HIV (n = 249)		Pregnant women diagnosed with HIV during pregnancy (n = 83)		p value
	n	%	n	%	n	%	
Age (years) in calendar year of pregnancy							
< 25	48	15	30	12	18	22	0.006
25–35	227	68	182	73	45	54	
≥ 35	57	17	37	15	20	24	
Migration background							
No migration background	185	56	172	69	13	16	< 0.001
First-generation	107	32	49	20	58	70	
Second-generation	40	12	28	11	12	14	
Urbanisation level^a^							
High	191	58	147	59	50	60	0.48
Middle/low	141	43	102	41	33	40	
Household income^b^							
Below the poverty line	47	14	15	6	32	39	< 0.001
Above the poverty line	263	79	224	90	39	47	
Unknown	22	7	10	4	12	14	
Household composition children							
Without child(ren)	115	35	95	38	20	24	< 0.001
With child(ren)	172	52	138	55	34	41	
Other/ unknown/no data	45	14	16	6	29	35	
Household composition partner^c^							
(Un)married couple (with or without children)	214	64	175	70	39	47	< 0.001
Single or single parent	62	19	32	13	30	36	
Other/unknown/no data	56	17	42	17	14	17	
Education							
Primary	37	11	24	10	13	16	< 0.001
Secondary/college/university	230	69	203	81	27	33	
Unknown	65	20	22	9	43	52	
Household income source							
Salaried employment/ self-employment/ assets	253	76	223	89	30	36	< 0.001
Social welfare benefits/student financial aids/pension benefits/ other/unknown	79	24	26	11	53	64	
Being employed	206	62	180	72	26	31	
Receipt of social welfare	29	9	11	4	18	22	
Gravidity before HIV diagnosis							
0	NA		NA		27	33	NA
≥ 1					56	67	
Stage of HIV at diagnosis							
Advanced diagnosis (CD4^+^ < 200 cells/mm^3^ or AIDS-defining event)	NA		NA		12	14	NA

NA: not applicable.

^a^ High is ≥ 1,500 addresses per square kilometre. Middle/low is < 1,500 addresses per square kilometre.

^b^ The poverty line is an income below 120% of the social minimum, which is the minimal amount of financial resources required to achieve a minimally acceptable lifestyle.

^c^ Includes both married and unmarried partners. Other includes in institutional households.

### Factors associated with HIV diagnosis during pregnancy

In the logistic regression model including household income (Table 3), being diagnosed with HIV during pregnancy was associated with a first- (aOR: 14.1; 95% CI: 6.45–30.6) or second-generation migration background (aOR: 5.38; 95% CI: 2.02–11.0), an income below the poverty line (aOR: 3.46; 95% CI: 1.28–9.34) and living in a single (parent) household (aOR: 3.46; 95% CI: 1.52–7.88). When education and employment status were included in the second logistic regression model instead of household income, first- and second-generation migration background and living in a single or single-parent household remained determinants of being diagnosed with HIV while pregnant, as were primary education (aOR: 5.12; 95% CI: 1.97–13.3) and being unemployed (aOR: 2.28; 95% CI: 1.14–4.52) (Table 3).

**Table 3:** Socioeconomic and demographic characteristics associated with an HIV diagnosis during pregnancy, the Netherlands, 2016–2023

Characteristics	Univariable model		p value	Multivariable model with household income^a^		p value	Multivariable model with education, social welfare and employment status^b^		p value
	OR	95% CI		Adjusted OR	95% CI		Adjusted OR	95% CI	
Age (years) in calendar year of pregnancy									
< 25	1		0.007	NA		NA	NA		NA
25–34	0.41	0.21–0.81							
≥ 35	0.90	0.41–2.00							
Migration background									
No migration background	1		< 0.001	1		< 0.001	1		0.001
First-generation migration	15.7	7.93–30.9		14.1	6.45–30.6		5.97	2.36–15.1	
Second-generation migration	5.67	2.35–13.7		5.38	2.02–11.0		5.00	1.83–13.7	
Urbanisation level^c^									
High	1		0.85	NA		NA	NA		NA
Middle/low	0.95	0.57–1.58							
Household income^d^									
Above poverty line	1		< 0.001	1		< 0.001	NA		NA
Below poverty line	12.3	6.07–24.7		3.46	1.28–9.34				
Unknown	6.90	2.79–17.1		4.92	2.19–11.4				
Household composition partner									
(Un)married couple (with or without children)	1		< 0.001	1		< 0.001	1		< 0.001
Single or single parent	4.20	2.29–7.72		3.46	1.52–7.88		6.84	3.08–15.2	
Other/unknown/no data	1.50	0.75–3.00		0.67	0.28–1.62		0.89	0.37–2.15	
Education									
Secondary/college/university	1		< 0.001	NA		NA	1		< 0.001
Primary	4.07	1.86–8.93					5.12	1.97–13.3	
Unknown	14.7	7.66–28.2					1.45	0.54–3.84	
Employment status									
Employed	1		< 0.001	NA		NA	1		< 0.001
Unemployed	5.72	3.33–9.82					2.28	1.14–4.52	
Household income source									
Salaried employment/ self-employment/ assets	1		< 0.001	NA		NA	NA		NA
Social welfare benefits/student financial aids/pension benefits/ other/unknown	15.2	8.29–27.7							

CI: confidence interval; NA: not applicable; OR: odds ratio.

^a^ In the multivariate model, education, employment status and household income source were excluded as they were collinear with household income. The candidate variable age was excluded as the p value was >0.05. The final model was adjusted for migration background, household income and household composition.

^b^ In the multivariate model, household income was excluded as it was collinear with education and employment status. The candidate variables age and household income source were excluded as the p value was >0.05. The final model was adjusted for migration background, household composition, education and employment status.

^c^ High is ≥ 1,500 addresses per square kilometre. Middle/low is < 1,500 addresses per square kilometre.

^d^ The poverty line is an income below 120% of the social minimum, which is the minimal amount of financial resources required to achieve a minimally acceptable lifestyle.

## Discussion

In this national study, we demonstrated that between 2016 and 2023, HIV diagnoses during pregnancy in the Netherlands accounted for nearly one in five (18%) of all HIV diagnoses in women aged 15–50 years. Women diagnosed during pregnancy were less often diagnosed with advanced-stage HIV and more often had an earlier negative HIV test, suggesting that a proportion of these diagnoses may have been recently acquired infections. First- and second-generation migration backgrounds and an income below the poverty line were more prevalent among women diagnosed with HIV during pregnancy than among pregnant women without HIV. Women diagnosed with HIV during pregnancy were less likely to share a household with a partner.

The proportion of advanced-stage HIV at the time of diagnosis was higher among non-pregnant than among pregnant women. Timely diagnosis of HIV is essential, as early initiation of ART not only reduces the risk of perinatal HIV transmission [14] but also improves maternal health outcomes. Antenatal HIV care delivered by a multidisciplinary team provides essential counselling and support for adjusting to an HIV diagnosis, initiating ART and preparing for the post-partum period [11]. Notably, more than half of the pregnant women diagnosed with HIV during pregnancy had a previously negative HIV test result, many of whom had been tested in the year before diagnosis. This may indicate that these women were already recognised as being at increased risk for HIV, or that prior testing was done during earlier pregnancies. While the indication for prior testing was not available, the observation that 63% of women diagnosed with HIV during pregnancy had a previous pregnancy supports the latter. Consistent with this observation, we found that women with a prior pregnancy more often had a previous negative HIV test result compared with first-time pregnant women.

We also identified two women who tested negative for HIV earlier in the same pregnancy before the HIV diagnosis. These women likely acquired HIV after, or shortly before, the first-trimester screening. To identify such missed or incident cases, repeat testing during pregnancy for women at increased risk could be considered. In addition, women at increased risk of HIV may benefit from pre-exposure prophylaxis (PrEP), although women are not currently considered a key population for PrEP [15]. Our findings support the reconsideration of PrEP access for women of reproductive age.

Among pregnant women with a prior pregnancy and a previous negative test, HIV was likely acquired in the relatively short interval between pregnancies. This may contribute to the lower occurrence of being diagnosed with advanced-stage HIV among pregnant women. Alternatively, the higher proportion of advanced disease among non-pregnant women may reflect differences in HIV testing, as non-pregnant women could have approached their healthcare provider who ordered an HIV test due to health issues, whereas pregnancy screening is universally offered irrespective of HIV related symptoms.

In the Netherlands, late diagnosis of HIV is highest among heterosexual men. Between 2022 and 2024, 62% of heterosexual men were diagnosed at a late stage compared with 32% of men who have sex with men [6]. This suggests that offering HIV screening to male partners of pregnant women may help identify women at risk and may also facilitate earlier HIV diagnosis in men. A study in London reported that 35% of male partners attending antenatal ultrasound examinations accepted screening for sexually transmitted infections, including HIV [16], indicating that antenatal care might provide a feasible setting to reach male partners of pregnant women [17].

In the Netherlands, HIV screening is offered to pregnant women in the first trimester. Our study included women classed as nulliparous despite evidence of previous pregnancies, likely reflecting early pregnancy loss or termination, who may not have undergone first-trimester antenatal HIV screening. Previous studies have reported higher or equal HIV prevalence rates among women undergoing termination of pregnancy compared with women in antenatal care [18] and have shown that HIV screening in these settings can be feasible and cost-effective in high income countries [18,19]. However, implementation may impose a considerable burden on women.

HIV screening is widely offered to pregnant women in European countries with participation rates above 95% in most countries [5,20]. In European countries, the pooled HIV prevalence among pregnant women was below 0.1% between 2001 and 2011 [21]. In our study, women diagnosed with HIV during pregnancy more often had an income below the poverty line compared with pregnant women in the general population. This is consistent with a previous study showing that pregnant women with HIV more often had lower incomes [22] and that women with a migration background were more often diagnosed late in pregnancy compared with women without a migration background [23]. The higher prevalence of low household income among pregnant women with HIV may partly be explained by their higher odds of living in single or single-parent households and being solely responsible for the household income. Other studies have also reported socioeconomic inequalities among individuals with HIV [24-26]. Advanced-stage HIV diagnosis was more common in neighbourhoods with lower socioeconomic position in Switzerland [24]. In the United States, individuals with HIV more frequently experienced unemployment, poverty and lack of housing compared with the general population [25] and in the Netherlands, women who lived in poverty were more likely to have detectable viral loads and be disengaged from care [26].

A strength of this study was the national setting, using data from the ATHENA observational cohort, which includes 97% of individuals receiving HIV care in the Netherlands. Combining data with Statistics Netherlands enabled an assessment of socioeconomic and demographic characteristics of pregnant women with and without HIV. Several limitations should be considered. First, not all women in the ATHENA cohort could be linked to Statistics Netherlands data. Women need to be an inhabitant with a postal code to be included in the data of Statistics Netherlands and unlinked women may be more likely to be unregistered or homeless and thus likely to be more socially vulnerable. Second, missing information related to socioeconomic characteristics were somewhat unevenly distributed between pregnant women with and without HIV. Consequently, the fact that not all women could be linked and missing information was not equally distributed may have led to an underestimation of the observed differences in socioeconomic characteristics. Third, distinguishing between late-stage and established HIV in pregnant women can be difficult because these stages are based on CD4^+^ cell counts and pregnancy-related haemodilution can temporary lower CD4^+^ cell counts by an average of 56 cells/mm^3^ [27]. This may have led to misclassification and could partly explain the observed higher occurrence of late-stage HIV at time of diagnosis among pregnant women compared with non-pregnant women.

## Conclusions

HIV diagnoses during pregnancy account for a substantial proportion of HIV diagnoses among women in the Netherlands, but pregnant women less often present with advanced-stage disease. Women with and without a migration background received an HIV diagnosis during pregnancy, showing the importance of universal antenatal HIV screening. The findings of this study support adapted prevention strategies, including preconception counselling and repeat HIV testing during pregnancy for women at increased risk and the reconsideration of PrEP access for women of reproductive age.

## Data Availability

All results presented here are calculated from non-public registry data from Statistics Netherlands (Centraal Bureau voor de Statistiek (CBS)), accessed through the Remote Access environment. All output from the Remote Access environment is independently verified by Statistics Netherlands to ensure data cannot be traced back to individuals. Statistics Netherlands was not involved in the calculation of any of the results presented. While the data are not publicly available, academic institutions can apply for access to the Remote Access environment through Statistics Netherlands (for additional information, see https://www.cbs.nl/en-gb/our-services/customised-services-microdata/microdata-conducting-your-own-research). ATHENA cohort data (without Statistics Netherlands data) used in this study are available upon reasonable request. Requests for data access can be made to: hiv.monitoring@amsterdamumc.nl. Requests will be reviewed on a case-by-case basis. Statistical information or data for separate research purposes from the ATHENA cohort can be requested by submitting a research proposal to SHM (https://www.hiv-monitoring.nl/english/research/research-projects/). The proposal will undergo review by representatives of SHM for evaluation of scientific value, relevance of the study, design, and feasibility, statistical power, and overlap with existing projects.

## References

[1] Joint United Nations Programme on HIV/AIDS (UNAIDS). Global report: UNAIDS report on the global AIDS epidemic 2012. Geneva: UNAIDS; 2012. Available from: https://www.unaids.org/en/resources/documents/2012/20121120_UNAIDS_Global_Report_2012

[2] De Cock KM Fowler MG Mercier E de Vincenzi I Saba J Hoff E Prevention of mother-to-child HIV transmission in resource-poor countries: translating research into policy and practice. JAMA. 2000;283(9):1175-82. 10.1001/jama.283.9.1175 10703780

[3] Cooper ER Charurat M Mofenson L Hanson IC Pitt J Diaz C Combination antiretroviral strategies for the treatment of pregnant HIV-1-infected women and prevention of perinatal HIV-1 transmission. J Acquir Immune Defic Syndr. 2002;29(5):484-94. 10.1097/00042560-200204150-00009 11981365

[4] Boer K Nellen JF Patel D Timmermans S Tempelman C Wibaut M The AmRo study: pregnancy outcome in HIV-1-infected women under effective highly active antiretroviral therapy and a policy of vaginal delivery. BJOG. 2007;114(2):148-55. 10.1111/j.1471-0528.2006.01183.x 17305888

[5] European Centre for Disease Prevention and Control (ECDC). Antenatal screening for HIV, hepatitis B, syphilis and rubella susceptibility in the EU/EEA. Stockholm: ECDC; 2016. Available from: https://www.ecdc.europa.eu/en/publications-data/antenatal-screening-hiv-hepatitis-b-syphilis-and-rubella-susceptibility-eueea

[6] van Sighem AI, Wit FWNM, Smit C, Jongen VW, Shalev N, Blomaard C. HIV monitoring Report 2025. Human Immunodeficiency Virus (HIV) Infection in the Netherlands. Amsterdam: Stichting HIV monitoring; 2025. Available from: https://www.hiv-monitoring.nl/nl/resources/monitoring-report-2025

[7] Op de Coul ELM Hahné S van Weert YWM Oomen P Smit C van der Ploeg KPB Antenatal screening for HIV, hepatitis B and syphilis in the Netherlands is effective. BMC Infect Dis. 2011;11(1):185. 10.1186/1471-2334-11-185 21718466 PMC3160399

[8] Mulder-Folkerts DKF van den Hoek JAR van der Bij AK Boer K Schutte MF Coutinho RA. Minder weigering van deelname aan HIV-screening door zwangeren in Amsterdam sedert de invoering van de standaard-HIV-screening--tenzij weigering (‘opting-out’-methode). [Less refusal to participate in HIV screening among pregnant women in the Amsterdam region since the introduction of standard HIV screening using the opting-out method]. Ned Tijdschr Geneeskd. 2004;148(41):2035-7. 15554003

[9] van der Ploeg CPB, Odijk JAM, van Lent M. Prenatale Screening Infectieziekten en Erytrocytenimmunisatie (PSIE) 2023. [Antenatal screening for infectious diseases and erythrocytes immunisation 2023]. RIVM; 2024. Dutch. Available from: https://www.pns.nl/documenten/proces-monitor-psie-2023 25898866

[10] van der Ploeg CPB, Odijk JAM, van Lent M. Prenatale Screening Infectieziekten en Erytrocytenimmunisatie (PSIE) 2022. [Antenatal screening for infectious diseases and erythrocytes immunisation 2022]. Amsterdam: RIVM; 2024. Dutch. Available from: https://www.pns.nl/documenten/proces-monitor-psie-2022 25898866

[11] Byrne L Short C-E Bamford A Bradshaw D Cheserem E Clarke E BHIVA guidelines on the management of HIV in pregnancy and the postpartum period 2025. HIV Med. 2025;26(11):1757-880. 10.1111/hiv.70091 41201115

[12] Boender TS Smit C Sighem AV Bezemer D Ester CJ Zaheri S AIDS Therapy Evaluation in the Netherlands (ATHENA) national observational HIV cohort: cohort profile. BMJ Open. 2018;8(9):e022516. 10.1136/bmjopen-2018-022516 30249631 PMC6169757

[13] Uitvoeringsinstituut Werknemersverzekeringen (UWV). Sociaal minimum. [Social minimum]. Amsterdam: UWV. [Accessed: 21 Nov 2024]. Dutch. Available from: https://www.uwv.nl/nl/toeslag/sociaal-minimum

[14] Mandelbrot L Tubiana R Le Chenadec J Dollfus C Faye A Pannier E No perinatal HIV-1 transmission from women with effective antiretroviral therapy starting before conception. Clin Infect Dis. 2015;61(11):1715-25. 10.1093/cid/civ578 26197844

[15] Wijstma E Jongen VW Boyd A de Vries HJC Schim van der Loeff MF Prins M Outcomes of a policy to prioritize populations with expected healthcare barriers for subsidized preexposure prophylaxis care in Amsterdam, the Netherlands. AIDS. 2025;39(1):75-84. 10.1097/QAD.0000000000004027 39352137

[16] Dhairyawan R Creighton S Sivyour L Anderson J. Testing the fathers: carrying out HIV and STI tests on partners of pregnant women. Sex Transm Infect. 2012;88(3):184-6. 10.1136/sextrans-2011-050232 22199134

[17] Clinical Info HIV.gov. Recommendations for the Use of Antiretroviral Drugs During Pregnancy and Interventions to Reduce Perinatal HIV Transmission in the United States. Rockville: Clinical Info HIV.gov. [Accessed: 12 Jun 2025]. Available from: https://clinicalinfo.hiv.gov/en/guidelines/perinatal/whats-new

[18] Creighton S Badham E Stacey L Reeves I. HIV testing in termination of pregnancy services. Sex Transm Infect. 2012;88(1):69. 10.1136/sextrans-2011-050210 22123162

[19] Filippidis P Francini K Jacot-Guillarmod M Mathevet P Lhopitallier L Cavassini M HIV testing in termination of pregnancy and colposcopy services: a scoping review. Sex Transm Infect. 2022;98(2):143-9. 10.1136/sextrans-2021-055111 34544889 PMC8862085

[20] European Centre for Disease Prevention and Control (ECDC). Antenatal screening approaches effective in preventing mother-child-transmission of HIV, hepatitis B, syphilis and rubella in vulnerable populations. Stockholm: ECDC; 21 Mar 2017. Available from: https://www.ecdc.europa.eu/en/publications-data/antenatal-screening-approaches-effective-preventing-mtct-hiv-hbv-syphilis-and

[21] Wu S Wang J Guo Q Lan H Sun Y Ren M Prevalence of human immunodeficiency virus, syphilis, and hepatitis B and C virus infections in pregnant women: a systematic review and meta-analysis. Clin Microbiol Infect. 2023;29(8):1000-7. 10.1016/j.cmi.2023.03.002 36921717

[22] Shoemaker ES Volpini K Smith S Loutfy M Kendall C. Equitable Timing of HIV Diagnosis Prior to Pregnancy: A Canadian Perspective. Cureus. 2021;13(7):e16691. 10.7759/cureus.16691 34466322 PMC8396133

[23] Favarato G Bailey H Burns F Prieto L Soriano-Arandes A Thorne C. Migrant women living with HIV in Europe: are they facing inequalities in the prevention of mother-to-child-transmission of HIV? The European Pregnancy and Paediatric HIV Cohort Collaboration (EPPICC) study group in EuroCoord. Eur J Public Health. 2018;28(1):55-60. 10.1093/eurpub/ckx048 28449111

[24] Gueler A Schoeni-Affolter F Moser A Bertisch B Bucher HC Calmy A Neighbourhood socio-economic position, late presentation and outcomes in people living with HIV in Switzerland. AIDS. 2015;29(2):231-8. 10.1097/QAD.0000000000000524 25396262

[25] Dasgupta S McManus T Tie Y Lin CY Yuan X Sharpe JD Comparison of Demographic Characteristics and Social Determinants of Health Between Adults With Diagnosed HIV and All Adults in the U.S. AJPM Focus. 2023;2(3):100115. 10.1016/j.focus.2023.100115 37790662 PMC10546490

[26] Jongen VW Boyd A Albers T Schat N Verhagen M van Zoest R Harnessing registry data to identify socio-demographic and socio-economic gaps in HIV care in the Netherlands. Nat Commun. 2025;16(1):10517. 10.1038/s41467-025-65512-6 41298373 PMC12658163

[27] Heffron R Donnell D Kiarie J Rees H Ngure K Mugo N A prospective study of the effect of pregnancy on CD4 counts and plasma HIV-1 RNA concentrations of antiretroviral-naive HIV-1-infected women. J Acquir Immune Defic Syndr. 2014;65(2):231-6. 10.1097/QAI.0000000000000013 24442226 PMC3898601

